# Global dose distributions of neutrons and gamma-rays on the Moon

**DOI:** 10.1038/s41598-023-40405-0

**Published:** 2023-08-15

**Authors:** Masayuki Naito, Hiroki Kusano, Satoshi Kodaira

**Affiliations:** National Institutes for Quantum Science and Technology (QST), 4-9-1 Anagawa, Inage, Chiba, 263-8555 Japan

**Keywords:** Environmental sciences, Planetary science, Risk factors, Astronomy and planetary science

## Abstract

Dose assessment on the lunar surface is important for future long-term crewed activity. In addition to the major radiation of energetic charged particles from galactic cosmic rays (GCRs), neutrons and gamma-rays are generated by nuclear interactions of space radiation with the Moon’s surface materials, as well as natural radioactive nuclides. We obtained neutron and gamma-ray ambient dose distributions on the Moon using Geant4 Monte Carlo simulations combined with the Kaguya gamma-ray spectrometer measurement dataset from February 10 to May 28, 2009. The neutron and gamma-ray dose rates varied in the ranges of 58.7–71.5 mSv/year and 3.33–3.76 mSv/year, respectively, depending on the lunar geological features. The lunar neutron dose was high in the basalt-rich mare, where the iron- and titanium-rich regions are present, due to their large average atomic mass. As expected, the lunar gamma-ray dose map was similar to the distribution of natural radioactive elements (^238^U, ^232^Th, and ^40^K), although the GCR-induced secondary gamma-ray dose was significant at ~ 3.4 mSv/year. The lunar secondary dose contribution resulted in an additional dose of 12–15% to the primary GCR particles. Global dose distributions on the lunar surface will help identify better locations for long-term stays and suggest radiation protection strategies for future crewed missions.

## Introduction

Human space activity is expected to extend to the Moon, Mars, and deep space in the next few decades. One of the major concerns of living in space is the exposure to radiation. The major radiation sources are galactic cosmic rays (GCRs), which consist of various energetic charged particles produced by high-energy explosion phenomena occurring in the galaxy, and solar energetic particles (SEPs), which consist mainly of protons resulting from a giant explosion in the Sun. Low-Earth orbits (LEO), where current space activities in the International Space Station (ISS) are ongoing, are partially protected from space radiation by geomagnetic fields. The lunar atmosphere is tenuous of ~ 10^−9^ bar^1^ and typical magnetic fields in the lunar environment is 10^−4^ G^2^, the order of 10^−3^ in comparison to the geomagnetic fields. The nearly non-existing lunar atmosphere and magnetic field allow space radiation to reach its surface. The effective dose equivalent was estimated at 420 mSv/year on the lunar surface by calculating the worst-case scenario during the minimum solar period^3^. The lunar radiation environment includes not only primary charged particles, but also secondary neutrons and gamma-rays, which are generated by the nuclear interactions of primary particles with the Moon’s surface materials, as well as natural radioactive nuclides such as ^238^U, ^232^Th, and ^40^K.

Space radiation dosimetry has been performed using various space programs^4–9^. Dose-equivalent rates of up to ~ 200 mSv/year in LEO and ~ 660 mSv/year in deep space have been reported. Numerical calculations have also been conducted to estimate the space radiation environment on the Moon, Mars, and deep space^10–13^. Although the dose measurement at the lunar surface was first conducted with a dosimeter on the Chang’E-4 lander^14^, measurements of the space radiation dose around/on the Moon and Mars are still very limited compared to those in LEO^14–16^.

While a large part of the exposure rate on the lunar surface is due to the primary charged GCR particles, secondary radiation of neutrons and gamma-rays produced by their nuclear interactions with surface materials, such as soil and rocks, also contributes partially to the radiation dose. Although some previous lunar exploration missions have measured neutrons and gamma-rays using remote sensing in planetary sciences based on lunar geology, dosimetry has yet to become a focus.

In this study, we estimated the dose distributions of neutrons and gamma-rays on the entire lunar surface from the Geant4 Monte Carlo simulations and the measurement dataset of Kaguya gamma-ray spectrometer (KGRS)^17,18^.

## Materials and methods

The ambient doses of neutrons and gamma-rays were calculated for selected lunar surface compositions. Subsequently, the global distribution of the ambient dose was derived using the KGRS observation data on the fast neutron flux and gamma-ray energy deposition rate. Here, direct dose measurement was not conducted due to restrictions in energy ranges of neutron and gamma-ray measurements, which substantially underestimates the measured doses. Therefore, we employed their relative variations normalized by the average of entire Moon to obtain the global dose distribution from the limited calculation points. The measured relative neutron fluxes and gamma-ray energy deposits at the Apollo and Luna sampling sites and the feldspathic highland terrain (FHT) were compared to the calculated neutron and gamma-ray ambient doses to derive the correlation between them. The measured values for the FHT were obtained by averaging over the northern far-side quarter of the lunar surface.

### Ambient dose on the lunar surface

The ambient doses on the lunar surface were calculated using the Geant4 toolkit^19–21^ version 10.5.1. The physics list of Shielding was employed by default. In addition, four other physics lists, FTFP_BERT_HP, QGSP_BERT_HP, QGSP_BIC_HP, and QGSP_INCLXX_HP, were used to estimate the uncertainty based on the Geant4 physics model.

20 m × 20 m × 10 m cuboids were modeled as the lunar materials with six different compositions, which were defined based on returned regolith samples from the Apollo and Luna missions and the feldspathic lunar meteorites in Table 1^22,23^. These regolith samples, which were mixed soils, were selected because they represented the average composition around the sampling site^24^. Feldspathic lunar meteorites were included as a representative composition of feldspathic highland terrain (FHT) for which there were no returned samples. The density of the modeled lunar surface was set to 1.6 g/cm^3^.

**Table 1:** The elemental compositions of Apollo (A) and Luna (L) returned samples, and FHT^22,23^. The numbers following the A or L denote their mission numbers, e.g., A11 means the Apollo 11 sample. The same numbering was employed in the following figures and tables.

Element	Weight fraction					
	A11	A12	A14	A16	L20	FHT
O	4.21 × 10^−1^	4.26 × 10^−1^	4.43 × 10^−1^	4.51 × 10^−1^	4.42 × 10^−1^	4.53 × 10^−1^
Na	3.26 × 10^−3^	3.41 × 10^−3^	5.19 × 10^−3^	3.41 × 10^−3^	2.60 × 10^−3^	2.60 × 10^−3^
Mg	4.76 × 10^−2^	6.15 × 10^−2^	5.67 × 10^−2^	3.62 × 10^−2^	5.73 × 10^−2^	3.26 × 10^−2^
Al	7.14 × 10^−2^	6.67 × 10^−2^	9.21 × 10^−2^	1.41 × 10^−1^	1.21 × 10^−1^	1.49 × 10^−1^
Si	1.96 × 10^−1^	2.15 × 10^−1^	2.23 × 10^−1^	2.10 × 10^−−1^	2.11 × 10^−1^	2.09 × 10^−1^
P	4.36 × 10^−4^	1.31 × 10^−3^	2.14 × 10^−3^	5.24 × 10^−4^	5.24 × 10^−4^	1.18 × 10^−−4^
S	1.10 × 10^−3^	8.00 × 10^−4^	1.00 × 10^−−3^	7.00 × 10^−4^	–	–
K	1.16 × 10^−3^	1.99 × 10^−3^	4.32 × 10^−3^	1.00 × 10^−3^	5.81 × 10^−−4^	2.24 × 10^−4^
Ca	8.58 × 10^−2^	7.36 × 10^−2^	7.79 × 10^−2^	1.09 × 10^−1^	1.03 × 10^−1^	1.16 × 10^−1^
Ti	4.50 × 10^−2^	1.62 × 10^−2^	1.02 × 10^−2^	3.54 × 10^−3^	2.94 × 10^−3^	1.32 × 10^−3^
Cr	2.05 × 10^−3^	2.60 × 10^−3^	1.37 × 10^−3^	7.59 × 10^−4^	1.30 × 10^−3^	6.57 × 10^−4^
Mn	1.63 × 10^−3^	1.63 × 10^−3^	1.08 × 10^−3^	5.42 × 10^−4^	8.52 × 10^−4^	4.88 × 10^−4^
Fe	1.23 × 10^−1^	1.28 × 10^−1^	8.16 × 10^−2^	4.23 × 10^−2^	5.67 × 10^−2^	3.42 × 10^−2^
Sm	1.30 × 10^−5^	1.60 × 10^−5^	3.00 × 10^−5^	6.00 × 10^−6^	3.10 × 10^−6^	1.10 × 10^−6^
Eu	1.77 × 10^−6^	1.75 × 10^−6^	2.50 × 10^−6^	1.20 × 10^−6^	9.10 × 10^−7^	7.80 × 10^−7^
Gd	1.70 × 10^−5^	2.00 × 10^−5^	3.50 × 10^−5^	8.00 × 10^−6^	4.00 × 10^−6^	1.30 × 10^−6^
Th	2.00 × 10^−6^	5.80 × 10^−6^	1.30 × 10^−5^	2.20 × 10^−6^	1.30 × 10^−6^	3.70 × 10^−7^
U	5.10 × 10^−7^	1.50 × 10^−6^	3.50 × 10^−6^	6.20 × 10^−7^	3.30 × 10^−7^	1.60 × 10^−7^

**Figure 1:**
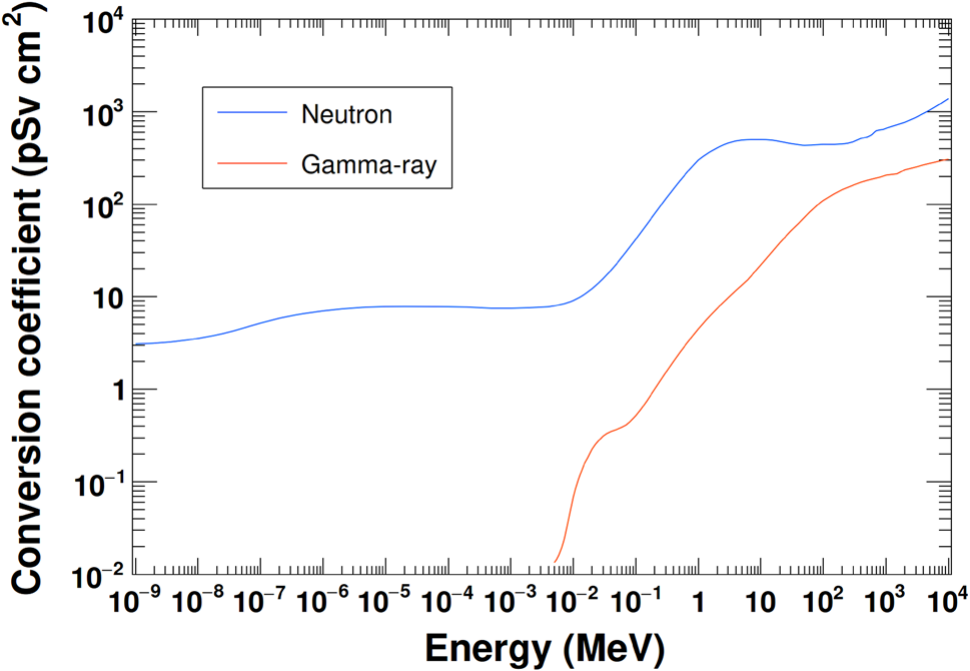
The ambient dose conversion coefficients for neutrons and gamma-rays^26^.

**Figure 2:**
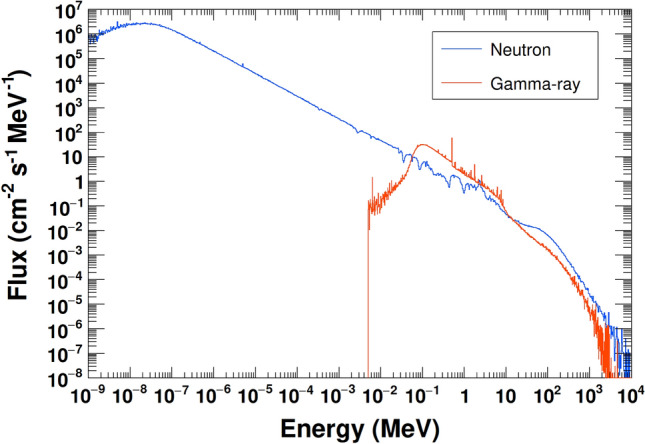
The calculated neutron and gamma-ray energy spectra for the Apollo 16 returned sample composition.

The modeled lunar surface was isotropically irradiated by GCR particles in the energy range of 10 MeV/n–100 GeV/n. GCR energy spectra were obtained using the DLR model^25^. The solar modulation parameter *W* was set to 7.45, which is the average value during the KGRS observation period. The neutron and gamma-ray fluxes emitted from a central 14 m × 14 m area were collected and converted to ambient doses using the conversion coefficients from ICRU report 95 (Fig. 1)^26^. The contributions of natural radioactive elements and their progeny nuclei were calculated separately, assuming that the nuclides ^40^K, ^232^Th, and ^238^U were uniformly distributed in the top 1.5 m of the lunar surface model. The natural gamma-ray flux and ambient dose were determined as described above. Figure 2 shows the neutron and gamma-ray spectra calculated using the composition of the Apollo 16 returned sample.

Table 2 shows the calculated ambient doses of neutrons and gamma-rays induced by the GCR H, He, and heavier ions (Li–Ni), based on the reference Apollo 16 sample composition. The GCR H and He contributed ~ 94% of the total ambient dose for both neutrons and gamma-rays. Thus, we derived the total ambient dose due to GCR particles by multiplying the ambient dose due to GCR H and He by a factor of 1.06 to consider heavy ion contributions for the other sample compositions.

**Table 2:** The calculated ambient dose contribution for each GCR component.

	Calculated ambient dose (mSv/year)	
	Neutrons	Gamma-rays
GCR H	48.7	2.70
GCR He	10.9	0.52
GCR Li-Ni	3.81	0.19
Total	63.4	3.42

### Kaguya gamma-ray spectrometer measurement

**Figure 3:**
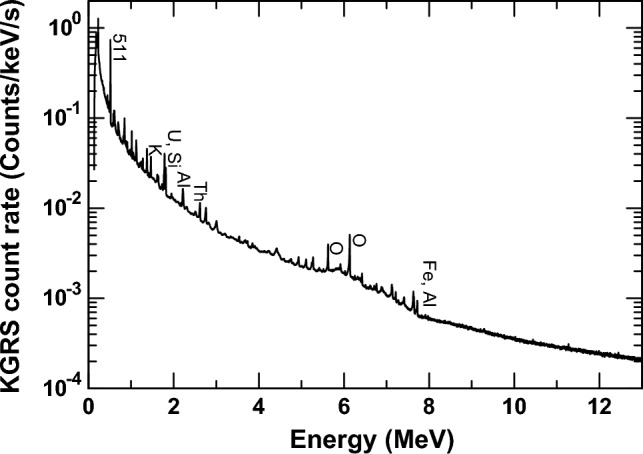
The gamma-ray energy spectrum observed by the KGRS for the entire Moon^28^. Some major gamma-ray peaks are labeled.

Kaguya (SELENE) is a Japanese lunar orbiter^27^, and the KGRS was observed for approximately 180 days between December 2007 and May 2009, acquiring gamma-rays from the lunar surface. The KGRS consisted of high-purity germanium (HPGe) as the main detector and a BGO anticoincidence scintillator. We employed the KGRS dataset obtained from low-altitude observations at 50 km from February 10 to May 28, 2009^28^. At this altitude, the spatial resolution of KGRS was 67 km × 67 km on the lunar surface. Figure 3 shows a gamma-ray energy spectrum measured by the KGRS in the energy range between 0.15 and 13 MeV for the whole surface. To obtain the regional variation in the gamma-ray energy spectrum, gamma-ray counts were accumulated every 2° × 2° in latitude and longitude using the moving average method in a window radius of 150 km.

Although Kaguya was not equipped with a neutron spectrometer, Hareyama et al.^29^ successfully obtained the global distribution of the fast neutron flux using specific gamma-ray peaks of KGRS, which were induced by inelastic scattering between fast neutrons and germanium nuclei. We employed the reported distribution of the fast neutron flux.

The relative gamma-ray energy deposit distribution was derived by integrating the product of the count rates *C*_*i*_ and energy *E*_*i*_ at each channel *i*:

1 $$\sum {C}_{i}\times {E}_{i}. $$

## Results

**Figure 4:**
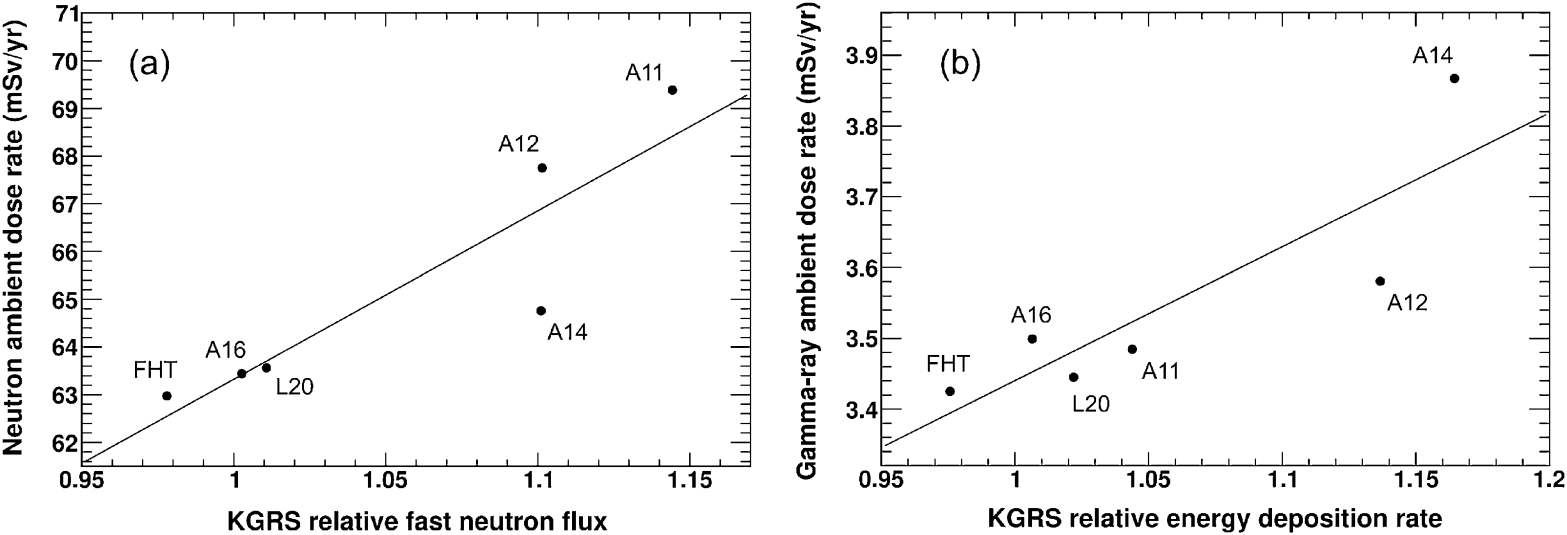
Relationship between the calculated ambient doses and the KGRS-measured (**a**) relative fast neutron flux and (**b**) relative gamma-ray energy deposition rate.

The calculated ambient doses, KGRS-measured relative fast neutron flux and gamma-ray energy deposition rate for the selected sampling sites are summarized in Table 3. Figure 4 show the relationship between the calculated ambient doses and the KGRS-measured relative fast neutron flux and gamma-ray energy deposition rate. The following linear correlations were obtained using least squares fitting:

**Table 3:** The calculated ambient doses, measured relative neutron flux^29^, and gamma-ray energy deposition rate at the sampling sites. The calculated gamma-ray ambient doses were divided into two components, the secondary gamma-rays induced by the GCR interactions and those emitted from the natural radioactive decays.

Sampling site	Calculated				Measured	
	Neutron (mSv/year)	Gamma-ray (mSv/year)			Relative fast neutron flux	Relative gamma-ray energy deposition rate
		Primary GCR	Natural	Sum		
A11	69.4	3.41	0.074	3.48	1.14	1.04
A12	67.8	3.38	0.202	3.58	1.10	1.14
A14	64.8	3.41	0.460	3.87	1.10	1.16
A16	63.4	3.42	0.082	3.50	1.00	1.01
L20	63.6	3.40	0.048	3.45	1.01	1.02
FHT	63.0	3.41	0.017	3.43	0.98	0.98

2 $$y\,\, (\text{mSv}/\text{year})=35.3x+28.1\,\, (\text{Neutron}) $$

3 $$y\,\, (\text{mSv}/\text{year})=1.89x+1.55,\,\, (\text{Gamma-ray}) $$

**Figure 5:**
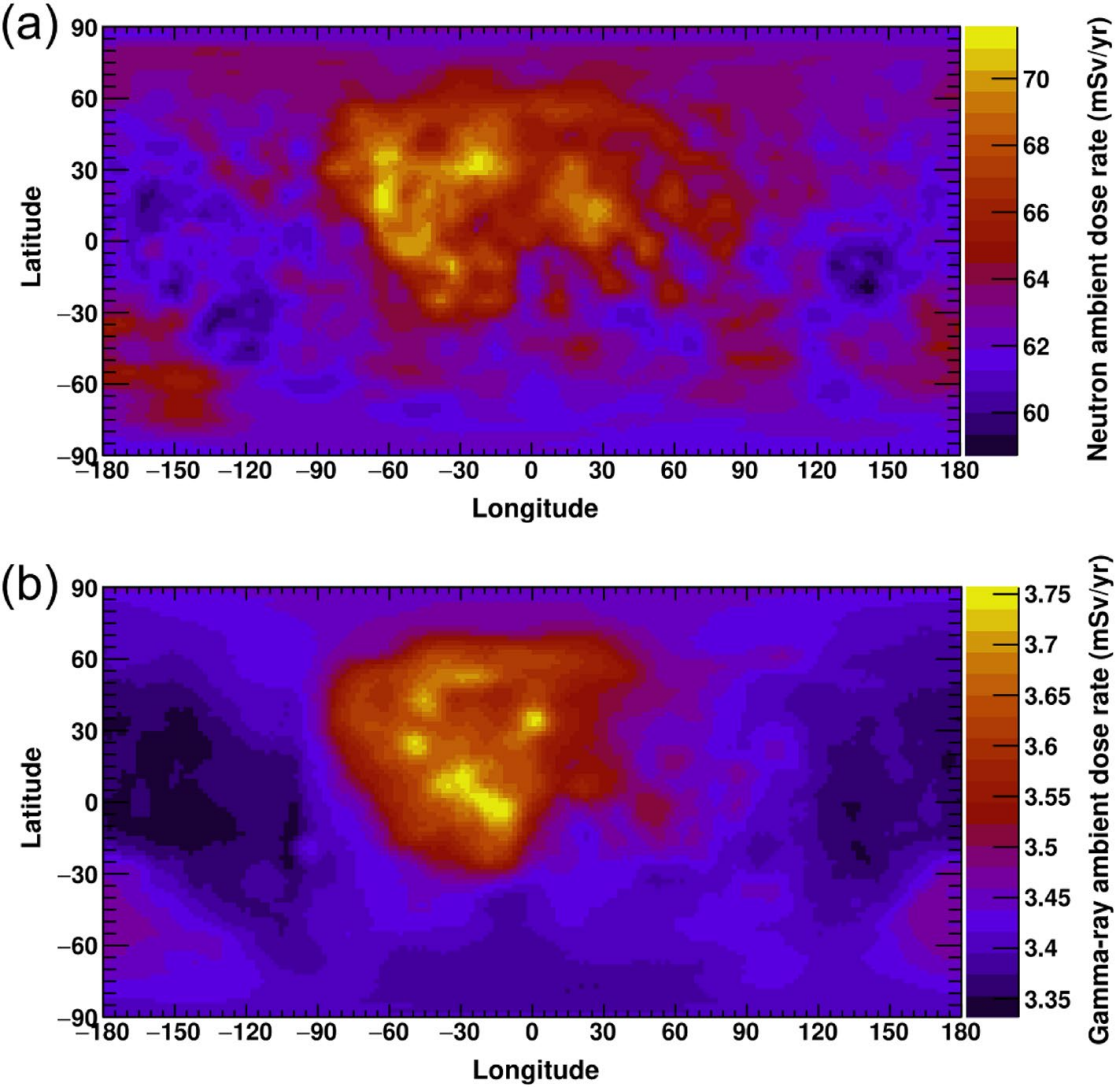
The (**a**) neutron and (**b**) gamma-ray ambient dose distributions on the entire lunar surface.

Although some data points did not fall on the linear correlations, this is likely due to differences between the calculations and KGRS measurements, such as energy ranges, uncertainty in the calculation physics model, and relatively large KGRS spatial resolution (67 km × 67 km). Nevertheless, good correlations were observed between the calculations and KGRS measurements for neutrons and gamma-rays, with linear correlation coefficients of 0.90 for neutrons and 0.87 for gamma-rays. The global ambient dose distributions of neutrons and gamma-rays on the lunar surface (Fig. 5) were derived using Eqs. (2) and (3).

Table 4 presents the differences in neutron and gamma-ray ambient doses among several physics models. While the gamma-ray contributions by the GCR protons were similar among the physics models, the other particle contributions varied by 27–45% depending on the models. The variation in the neutron dose caused by the GCR protons was the most significant. The Shielding physics list employed to obtain the above correlation provided the most significant ambient dose of GCR H and a small ambient dose of GCR He. This dependence may provide additional uncertainty to the results depending on the calculation models.

**Table 4:** The calculated ambient doses of neutrons and gamma-rays induced by GCR H, He, and their sum with various Geant4 physics models.

Physics lists	Calculated ambient dose (mSv/year)					
	Neutrons			Gamma-rays		
	GCR H	GCR He	Sum	GCR H	GCR He	Sum
Shielding	48.7	10.9	59.6	2.70	0.52	3.22
FTFP_BERT_HP	47.9	13.4	61.3	2.68	0.71	3.39
QGSP_BERT_HP	46.6	13.3	59.9	2.69	0.72	3.41
QGSP_BIC_HP	34.7	11.0	45.8	2.66	0.71	3.36
QGSP_INCLXX_HP	33.5	10.5	44.0	2.71	0.68	3.39
Max/Min	1.45	1.27	1.39	1.02	1.39	1.06

## Discussion

The neutron and gamma-ray ambient doses varied in the ranges of 58.7–71.5 mSv/year and 3.33–3.76 mSv/year, respectively. The variations through the entire Moon were ~ 18% for neutron and ~ 12% for gamma-ray while the total errors in the relative neutron fluxes and gamma-ray energy deposition measured by KGRS were ~ 3%^29^ and < 1%, respectively. Thus, the dose variations depending on the lunar surface geological aspects are significantly large. Note that our calculations assumed a KGRS observation period during the minimum phase of solar activity. Therefore, these dose rates represented the worst cases in the solar cycle. Figure 5 a and b provide the specific dose distributions depending on the geological features, whereas the previous measurements and calculations were limited to a specific location or an average on the Moon^3,10,14,30^. The mean neutron dose rates were 20–30% higher than those in previous calculations^3,10^. This was attributed to differences in the dosimetric definitions employed. The ambient dose was newly defined by ICRP and ICRU as the maximum conversion coefficient of the effective dose under various irradiation conditions^26^.

The neutron and gamma-ray dose distributions were consistent with the regional variation of the surface elements obtained from previous lunar gamma-ray measurements^31–38^. Iron and titanium^31–33^, which are rich in basaltic mare materials, distribute similar to neutron doses. Generally, nuclear fragmentation between the GCR particles and lunar surface materials is the major reaction that produces neutrons on the Moon. The fragmentation cross-section increases by a factor of *A*^2/3^, where *A* is the atomic mass of the material^39^. In addition, particle interactions with heavier nuclei produce larger numbers of secondary particles. Thus, the fast neutron distribution depends on the average atomic mass^40^. Since iron and titanium are major heavy elements in lunar materials, the neutron dose distribution was similar to their ones. Geological maps of the natural radioactive elements potassium, thorium, and uranium^31,34–38^ are similar to those of the gamma-ray dose map. While the dose due to GCR secondary gamma-rays was almost constant at ~ 3.4 mSv/year among the lunar samples, the natural radioactive gamma-ray doses ranged from 0.017 to 0.46 mSv/year, which defined the regional variation of the gamma-ray ambient dose on the lunar surface. The contribution of GCR secondary gamma-rays sufficiently occupies the lunar gamma-ray ambient dose, whereas regional variation is attributed to natural radioactive elements.

A dose rate of ~ 500 mSv/year due to primary GCR particles was reported in the solar minimum by a lunar orbiter and lander^14,30^. Our results imply that lunar secondary doses provide an additional dose of 12–15% of the primary GCR dose, depending on the lunar region. The absorbed dose rate due to neutral particles (including gamma-rays) was measured to be ~ 27 mGy/year in silicon (corresponding to 36 mGy/year in water with a conversion factor of 1.33)^14^ without any dosimetric weighting factors. The mean radiation weighting factor for our calculated neutron doses was 8.2–8.8 for the selected sampling sites. When we simply divide the ambient dose rate by the mean radiation weighting factor, the absorbed dose rate is estimated as 6.90–8.41 mGy/year for neutrons. Even though there are contributions from the gamma-ray absorbed dose, this value is much smaller than the measurement by a factor of ~ 4–5. Comparison of neutron doses between our evaluation and the Chang’E-4 measurement is summarized in Table 5. This may imply that there is still a disparity in the neutron measurements and calculations owing to the differences in detection media and evaluation methods, because the previous lunar neutron calculations also provided smaller neutron dose levels^3,10^. The dose contribution of GCR secondary particles should be considered for the space crew’s career dose limit. Strategic radiation protection for secondary particles, in addition to primary GCR particles, is required for future crewed lunar and planetary missions.

**Table 5:** Comparison of lunar neutron doses between our evaluation and the Chang’E-4 measurement^14^.

	Chang’E-4 measurement^14^		This work
Materials	In silicon	In water (conversion factor; 1.33)	In human tissue
Radiation weighting factor	n.d	n.d	8.2–8.8
Absorbed dose (mGy/year)	~ 27	~ 36	6.90–8.41
Ambient dose	–	–	58.7–71.5

The mean annual dose by natural terrestrial sources on the Earth is ~ 0.48 mSv/year with a typical range of 0.3–1 mSv/year^41^. The mean natural source exposure on the Moon (~ 0.037 mSv/year) is much lower than that on Earth by a factor of 10. In fact, previous lunar missions have reported differences in the abundance of natural radioactive nuclides on the Earth and Moon surfaces^31,36,37,41^ despite the belief that the Moon originates from Earth^42^. One possible explanation is that the igneous activity of the Earth transports incompatible natural radioactive nuclides to the surface, whereas lunar activity is already inactive. The lunar terrestrial dose level is very low and not significantly different from that on Earth. If the GCR exposure can be sufficiently relieved, a similar safe radiation environment can be achieved on the Moon.

## Conclusion

Global lunar dose distributions of neutrons and gamma-rays were evaluated by combining Monte Carlo simulations with the Kaguya gamma-ray spectrometer dataset. The neutron and gamma-ray doses varied in the range of 58.7–71.5 and 3.33–3.76, respectively, having distributions relating to lunar geologic features. The neutron dose distribution was similar to that of lunar mare materials, which have a large average atomic mass owing to their fast neutron production rates. The gamma-ray dose variation depended on lunar natural radioactive elements, whereas the GCR secondary gamma-ray dose was significant. Although the GCR secondary dose rates were smaller than the primary GCR dose rates, they contributed an additional 12–15% dose depending on the lunar region. These rates will not be negligible for future human space activities. Radiation protection against these secondary particles is also important, as is that against the primary GCR particles. Lunar global dose maps will help identify better locations for long-term stay and suggest radiation protection strategies for future crewed missions.

## Data Availability

The data sets generated in this study are available from the corresponding author upon reasonable request. There are no restrictions on data availability.
